# Purple Pitcher Plant (*Sarracenia rosea*) Dieback and Partial Community Disassembly following Experimental Storm Surge in a Coastal Pitcher Plant Bog

**DOI:** 10.1371/journal.pone.0125475

**Published:** 2015-04-13

**Authors:** Matthew J. Abbott, Loretta L. Battaglia

**Affiliations:** Department of Plant Biology and Center for Ecology, Southern Illinois University, Mail Code 6509, Carbondale, IL, 62901, United States of America; Ecole Pratique des Hautes Etudes, FRANCE

## Abstract

Sea-level rise and frequent intense hurricanes associated with climate change will result in recurrent flooding of inland systems such as Gulf Coastal pitcher plant bogs by storm surges. These surges can transport salt water and sediment to freshwater bogs, greatly affecting their biological integrity. Purple pitcher plants (*Sarracenia rosea*) are Gulf Coast pitcher plant bog inhabitants that could be at a disadvantage under this scenario because their pitcher morphology may leave them prone to collection of saline water and sediment after a surge. We investigated the effects of storm surge water salinity and sediment type on *S*. *rosea* vitality, plant community structure, and bog soil-water conductivity. Plots (containing ≥1 ramet of *S*. *rosea*) were experimentally flooded with fresh or saline water crossed with one of three sediment types (local, foreign, or no sediment). There were no treatment effects on soil-water conductivity; nevertheless, direct exposure to saline water resulted in significantly lower *S*. *rosea* cover until the following season when a prescribed fire and regional drought contributed to the decline of all the *S*. *rosea* to near zero percent cover. There were also significant differences in plant community structure between treatments over time, reflecting how numerous species increased in abundance and a few species decreased in abundance. However, in contrast to *S*. *rosea*, most of the other species in the community appeared resilient to the effects of storm surge. Thus, although the community may be somewhat affected by storm surge, those few species that are particularly sensitive to the storm surge disturbance will likely drop out of the community and be replaced by more resilient species. Depending on the longevity of these biological legacies, Gulf Coastal pitcher plant bogs may be incapable of fully recovering if they become exposed to storm surge more frequently due to climate change.

## Introduction

Gulf Coast pitcher plant (GCPP) bogs are highly diverse, nutrient poor systems (nitrogen, phosphorus, and potassium are the limiting nutrients) that are native to the southeastern region of the United States. It is not uncommon to find more than 40 species of plants in a 1 m^2^ plot in GCPP bogs [1], and thus they are considered to be some of the most diverse plant communities in North America [2]. Gulf Coast pitcher plant bogs, which once covered approximately 2,935 km^2^ in the southeastern portion of the United States prior to European settlement, have declined to just a mere 3% of their original extent due to anthropogenic disturbances such as logging, habitat fragmentation, and fire suppression [3]. Since a large majority of GCPP bogs occur close to the Gulf coastline (historically, the largest expanses were found within present day coastal counties [3]), climate change may exacerbate this already steady decline. Rising sea levels [4–7] and a higher frequency of intense hurricanes predicted with climate change [8–11] will likely have dramatic effects on coastal plant communities along the coastline of the Gulf of Mexico [12]. Many of the species residing in coastal wetlands must adapt to or migrate away from increasing salt water exposure associated with higher sea levels and farther reaching hurricane-induced storm surges [4, 13]. Historically, only during rare, exceptionally strong hurricane events did storm surges ever reach far enough inland to affect GCPP bogs; however, higher sea levels combined with potentially more intense hurricanes [8–11] are likely to produce storm surges that will reach inland communities such as GCPP bogs more regularly [4].

Storm surges can have dramatic effects on plant communities because they transport salt water, sediment, and other forms of debris inland. The elevated salinity levels can lead to short term mortality of salt-intolerant species [14] and deposits of debris can initiate shifts in community structure as well [15]. In a comparison of plant community responses to experimental storm surge treatments across an estuarine gradient, Tate and Battaglia [15] found that the degree of community response to the treatments was positively related to its distance from the coast [15–16]. Thus, since GCPP bog communities are on the inland end of this transitional gradient (where salinity levels are low and storm-driven sediment redistribution is uncommon) [3], the impact of storm surge on these communities is likely to be dramatic. Salt water flooding, sedimentation, or a combination of the two are therefore expected to drive shifts in community structure as intolerant GCPP bog species are lost and species resilient to these disturbances increase in abundance [4, 14].

Of the GCPP bog species that would likely have the most difficulty recovering from storm surge disturbance, the purple pitcher plant, *Sarracenia rosea*, may be the most sensitive. The short, stout, open-lid morphology of *S*. *rosea* leaves (an adaptation for the collection of precipitation [17]) may make it quite prone to accumulation of salt water and sediment within its pitcher-shaped leaves (hereafter referred to as ‘pitchers’) after a surge floods an area and recedes. Once considered to be part of the wide ranging *Sarracenia purpurea* population, the southern variety (i.e. *Sarracenia purpurea var*. *burkii* D.E. Schnell, which is listed as threatened in Florida) was identified as its own species, *Sarracenia rosea*, in 1999 [18]. The native range of *S*. *rosea* is mainly limited to the coastal counties of Alabama, Mississippi, Georgia, and the Florida panhandle [18]. With such a limited range, this species is particularly vulnerable to extinction via over-collection, land use change, and now, perhaps, farther reaching storm surges. Since *S*. *rosea* are not normally exposed to high salinity, residual salt water within the pitchers may cause immediate damage to the plants and could perhaps kill the inquiline communities on which the *S*. *rosea* depend for digestion of prey material. In addition, if the salt water does not immediately kill the *S*. *rosea*, sediments deposited in the pitchers might inhibit nutrient absorption from captured prey, preventing the plants from maintaining normal growth. Therefore, if a storm surge floods a GCPP bog *S*. *rosea* may be the first to drop out from the community.

Though it has been well documented that hurricane disturbances can lead to extensive mortality and assemblage shifts in coastal plant communities [4, 14–15], the effects of storm surges on plant communities within GCPP bogs have not yet been addressed. In this study, we experimentally applied storm surge treatments to GCPP bog communities and examined the effects of the treatments on *S*. *rosea* cover, overall plant community composition, and soil-water conductivity. We hypothesized that saline water intrusion and sedimentation from storm surge would (1) result in significant decreases in *S*. *rosea* cover; (2) cause shifts in plant community composition; and (3) result in elevated soil-water conductivity within GCPP bog soils.

## Materials and Methods

### Ethics Statement

The study was conducted on federal property on Eglin Air Force Base (AFB). All permits necessary for conducting field work were obtained from the Eglin AFB 96th Test Wing Range Configuration Control Committee. No protected species was destructively sampled from the property in this study.

### Study Site and Experimental Design

The GCPP bog used in this study was located in coastal wet pine savanna on Eglin AFB property (30° 29’ 0” N, 86° 31’ 31” W) in northwestern Florida (USA). We were constrained to this site because it was the only location at Eglin AFB that both supported a sufficiently high population of *Sarracenia rosea* and was within 300 meters of East River, the limit for effective pumping of water treatments to plots (see treatment description below). As is characteristic of typical fire-maintained, wet pine savannas, the overstory vegetation is composed mostly of longleaf (*Pinus palustris*) and slash (*Pinus elliottii*) pines, while the understory is characterized by a diverse assemblage of forbs, sedges and grasses [19]. The soils are nutrient-poor, sandy and acidic. Based on data from a nearby weather station at Pensacola Regional Airport [20], this area has an average annual temperature of 19.9° C and average annual rainfall total of 163 cm. However, the study site received only 103.2 cm of rainfall from September 2010 to August 2011 due to a regional drought.

This experiment was conducted during official hurricane season [20]. On August 19, 2010, eighteen 0.38 m^2^ plots (size corresponds to area of bottomless tank opening (described below))—each containing at least one ramet of *Sarracenia rosea*—were established. Each plot was then randomly assigned to one of three ‘sediment treatments’ and one of two ‘water treatments’ (three replicates for each treatment combination). One-third of the plots received sandy surface sediment taken from the immediate vicinity of the treated plots (hereafter referred to as ‘local’ sediment), one-third of the plots received a 2:1 surface sediment mixture of marine sand (collected from East Bay) and riverine silt (collected from East River) (hereafter referred to as ‘foreign’ sediment), and one-third of the plots received no sediment. A total of 18.9 liters of sediment was used to achieve a 5 cm sediment depth application for both the local and foreign sediment treatments [21]. Half of the plots were then flooded with 378.5 L of salt water and the other half was flooded with the same amount of fresh water. Storm surge simulations were accomplished by pumping pre-mixed sea water substitute (28 ppt–mixed with *Instant Ocean* sea salt (Aquarium Systems, Mentor, OH)) into the plots designated as ‘salt water’ plots and pumping fresh water into the ‘fresh water’ plots. Water for both the fresh water treatments and salt water treatments was taken from the nearby East River. A bottomless tank with a 0.38 m^2^ bottom frame was driven ~10 cm into the soil to create a seal and then held in place over each plot as the water was pumped into an overhanging container full of the sediment mix; the slurry was then poured onto the plot. The bottomless tank was kept on the plot until the water from the water/sediment slurry fully percolated through the soil surface (typically less than five minutes).

The three sediment treatments were meant to simulate the various forms of sediments that may accompany a storm surge. For instance, depending on the location of a particular site in relation to the hurricane wind patterns, a site may receive sediment taken from around the same vicinity of where it is eventually deposited [22]. If the sediment deposit originates from a ‘local source’, the sediment could be considered functionally ‘neutral’ because it would not be adding anything new (e.g., salt) to the soil. In most cases, however, storm surge sediment deposits originate (at least in part) from foreign sources [22]. Some of these allochthonous sediments could be characteristically different (i.e., salinity, nutrients, texture, etc.) from the soils on which they are deposited. The foreign sediment treatments were thus used in addition to local sediment treatments to determine if burial by foreign sediment has a greater effect than burial by local sediment (indicating a possible interaction of burial and sediment type) on the plant community. To simulate cases when little to no sediment accompanies a storm surge (or new sediment deposits are immediately washed away) [23], the rest of the plots received no sediment with the surge treatments. Finally, half of the plots were flooded with salt water to simulate saline surges, and the other half were flooded with fresh water to control for any flooding effect. Since it was not possible to apply the sediment slurry treatments uniformly across the plots without water, we did not have non-flooded control plots (we acknowledge, though, that the inclusion of true untreated controls would have strengthened the experiment). The fresh water/no sediment treated plots control for sediment treatment effects.

### Data Collection

To assess both short and longer term treatment effects on *S*. *rosea* mortality, we evaluated percent cover of *S*. *rosea* before treatment application and approximately three weeks, three months, one year, and two years after treatments. Plant community composition surveys (i.e., percent cover of each species (nomenclature follows that used by Clewell [24]) present as estimated and viewed from above) were also conducted before treatment application and approximately one and two years after treatments. Three 10 cm deep soil core samples were randomly collected (and subsequently homogenized) from each plot with a 2 cm diameter soil corer before treatment application and approximately three weeks, three months, and one year after treatments. The soil samples were taken at a minimum of 10 cm away from the *S*. *rosea* to minimize potential root damage. The soils were analyzed for soil water conductivity (SWC) by taking 22 g of soil for each plot and sampling date, mixing them with 110 mL of deionized water in separate glass flasks, and then placing them on an orbital tabletop shaker (New Brunswick Scientific Co., Inc., Edison, NJ) at 200 rpm for 1 hour. After shaking, the samples were immediately measured for SWC with an electrical conductivity meter (EcoSense EC300; YSI Inc., Yellow Springs, OH). Soil-water conductivity was used as a surrogate for measuring salinity because the electrical conductivity meter (which also measures salinity) gave a more accurate measurement of SWC than salinity [25].

### Statistical Analyses

Prior to performing any analysis of variance (ANOVA) statistics, all data were checked for conformity to ANOVA’s assumptions of normality and homoscedasticity using the Kolmogorov-Smirnov test and Levene’s test, respectively. These analyses revealed that all the datasets failed to meet either one or both of these assumptions; log transformation resolved the issue. Two-way repeated measures mixed model ANOVA (water treatment × sediment treatment) was used to compare change in percent cover of *S*. *rosea* across time and treatments and it was also used to compare pre- and post-treatment SWC (SAS, version 9.1). The water treatment was treated as a random effect and the sediment treatment was treated as a fixed effect in the analyses. Three-way mixed model ANOVA (water treatment × sediment treatment × species) was used to analyze for differences in percent cover change between *S*. *rosea* and three common neighboring species, one and two years after the initiation of the experiment (SAS, version 9.1). The water treatment was a random effect, while sediment and species were fixed effects. Species used in this three-way comparison were chosen because they were present in all or most of the plots before the study began.

To statistically test whether plant community composition differed across treatment groups over time [26], we used repeated measures permutational multivariate analysis of variance (PERMANOVA) and post-hoc pairwise comparisons, as appropriate, using PRIMER software [27]. PERMANOVA, a non-parametric, multivariate technique, uses permutation with pseudo-F ratios to generate p-values [26]. These analyses were based on Bray-Curtis dissimilarity values of species abundances (i.e., percent cover as estimated and viewed from above) standardized to species’ maxima. We ran 9999 permutations per test.

PRIMER was also used to calculate species richness (S), Pielou’s evenness index (J’), Shannon’s diversity index (H’), and Simpson’s index of dominance (λ) for each plot, and these calculations were compared across treatments and time using two-way repeated measured mixed model ANOVA (SAS, version 9.1). Finally, separate Indicator Species Analyses (ISA; [28]) were conducted for each community sampling date using PC-ORD to determine which species, if any, exhibited fidelity and constancy with any particular water treatment or sediment treatment (or a combination of the two). *S*. *rosea* was omitted from the ISA because plots had been intentionally established around the *S*. *rosea* during the initial setup of the experiment.

## Results

### Storm Surge Treatment Effects on *Sarracenia rosea*

**Fig 1:**
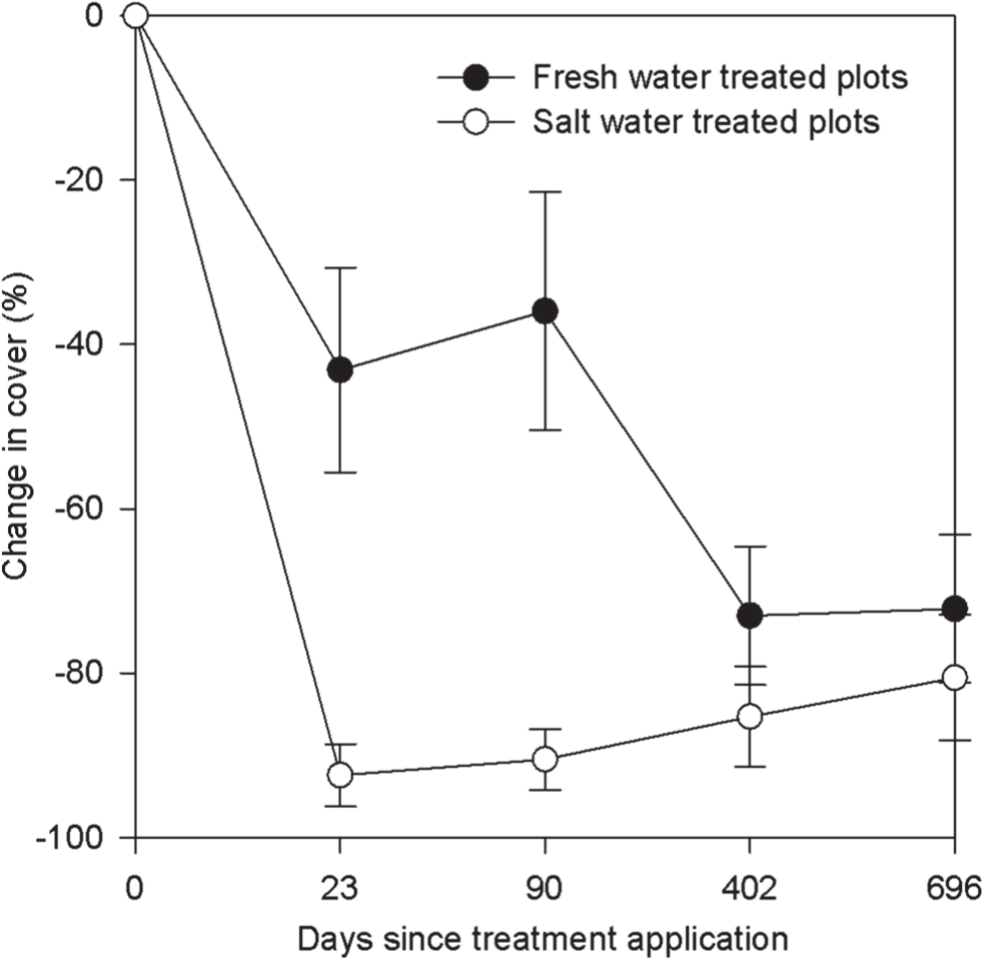
Effects of storm surge treatments on *Sarracenia rosea* over time. Line graph comparing *Sarracenia rosea* percent cover change across storm surge water treatments over time.

The *S*. *rosea* in this study exhibited sensitivity to the storm surge flooding treatments. Our results revealed that for change in percent cover of *S*. *rosea*, there was no sediment treatment effect, but there was a significant water treatment effect (F_1, 25.8_ = 15.19, p = 0.001), a significant effect of time since treatment (F _4, 30.2_ = 219.59, p < 0.001), and a significant water treatment × time interaction (F _4, 30.2_ = 8.36, p < 0.001). *S*. *rosea* decreased significantly in both the fresh water (t = -16.20, p < 0.001) and salt water (t = -23.65, p < 0.001) treated plots between day 0 and day 23, but the decrease was significantly greater in the salt water treated plots (t = -23.65, p = 0.008) and remained greater through the 90^th^ day (Fig 1; t = 4.61, p = 0.002). Though the salt water appeared to have a significant effect on the *S*. *rosea*, there were no observed differences in SWC between treatments twenty three days after the treatments or beyond. There was a significant time effect (F_1, 16.8_ = 46.22, p < 0.001); however, natural variation in soil moisture (e.g., via precipitation fluctuations) was likely responsible for the observed fluctuations in ionized solute concentrations.

A fire and drought occurred during the second growing season and may have masked any storm surge legacy effects that would have otherwise been still visible the following year. The stresses induced by one of or a combination of these disturbances likely drove *S*. *rosea* declines in all of the plots (across both the fresh water and salt water treated plots) to similarly low levels; plants remained in this state through the end of the experiment (Fig 1). The fire was scheduled under prescription and burned all but one foreign sediment/salt water treated plot (excluded from the statistical analyses) at the beginning of the 2011 growing season. The drought affected the majority of the Gulf Coast region, and the panhandle of Florida specifically received 58.8 cm below average rainfall between September 2010 and August 2011 [20].

### Storm Surge Treatment Effects on Plant Community Composition

The storm surge treatments appeared to have a slight effect on GCPP bog plant community composition. The repeated measures PERMANOVA comparing assemblage structure between treatments over time revealed a significant time effect (pseudo-F_2,22_ = 12.696, p < 0.001; Table 1) and a significant water treatment × sediment treatment × time interaction (pseudo-F_4,22_ = 1.633, p = 0.021; Table 1). However, despite the three-way interaction being significant, the pair-wise comparisons did not reveal any statistically significant or ecologically interpretable differences (i.e., p ≥ 0.05). A couple of pairwise comparisons did approach significance: the salt water/foreign sediment treated plots in 2010 were marginally different, compositionally, than the same plots in 2011 (t = 2.126, p = 0.099) and the fresh water/local sediment treated plots in 2010 were marginally different, compositionally, than the same plots in 2011 (t = 1.752, p = 0.096). However, there is no evidence that these groups changed over time in a direction different than the other treatment groups, and thus these effects are likely just a reflection of the strong main effect of time. Unrelated to the main effect of time, in 2012, composition of plots treated with fresh water diverged between foreign vs. local sediment treatments (t = 1.259, p = 0.097).

**Table 1:** PERMANOVA tests results for plant community composition differences between water treatments, sediment treatments, and years.

Factor	df	SS	MS	Pseudo-F	p(perm)
Water Treatment	1	5279.8	5279.8	1.0706	0.392
Sediment Treatment	2	11397	5698.4	1.1555	0.271
Year	2	20147	10073	12.696	0.001
Water Treatment ×Sediment Treatment	2	8837.1	4418.6	0.8960	0.640
Water Treatment ×Year	2	1297.2	648.58	0.8174	0.660
Sediment Treatment ×Year	4	2916.4	729.1	0.9189	0.593
Observation(Water Treatment ×Sediment Treatment)	11	54246	4931.5	6.2152	0.001
Water Treatment ×Sediment Treatment ×Year	4	5182.4	1295.6	1.6328	0.021
Residual	22	17456	793.46		
Total	50	1.27E+05			

Some species appeared more tolerant than others to certain aspects of storm surge. For instance, *Rhexia alifanus* was a significant indicator of fresh water treated plots one year after treatments (and remained a marginally significant indicator the following year) because it was found less often and less abundant in the salt water treated plots (Table 2). *Dichanthelium dichotomum* was present in all the plots, across all the treatments, but was most abundant in plots that received only fresh water and no sediment one year after treatments and continued to be most abundant in plots that received no sediment the following year (Table 2). Some species appeared to actually benefit from storm surge sediment deposits. *Rhexia lutea* was a significant indicator of foreign sediment treated plots one year after treatments; *Ilex glabra* was a significant indicator of local sediment treated plots one year after treatments; and *Anthaenantia rufa* was a significant indicator of foreign sediment treated plots two years after treatments (Table 2). It is most unlikely that there were any viable seeds from foreign species (i.e., not native to GCPP bogs) in the sediment treatments given the sources from which they were collected; further, all species accounted for in the floristic surveys are native to GCPP bogs and present in the pre-surge communities.

**Table 2:** Selected results (based on significance) from indicator species analysis (ISA) using percent cover data for 2011 and 2012 plant community composition.

Year	Species	Growth form	Treatment	Indicator value	p-value*
2011	*Rhexia alifanus*	Forb/Herb	Fresh water	69.9	0.018
2011	*Rhexia lutea*	Forb/Herb	Foreign sediment	60.0	0.030
2011	*Ilex glabra*	Tree/Shrub	Local sediment	66.7	0.013
2011	*Dichanthelium dichotomum*	Graminoid	Fresh water/no sediment	24.2	0.020
2012	*Ilex glabra*	Tree/Shrub	Local sediment	60.8	0.026
2012	*Dichanthelium dichotomum*	Graminoid	No sediment	45.9	0.031
2012	*Anthaenantia rufa*	Forb/Herb	Foreign sediment	12.10	0.026

*Species with p-values ≤ 0.05 are considered to be indicator species for their respective treatment.

**Fig 2:**
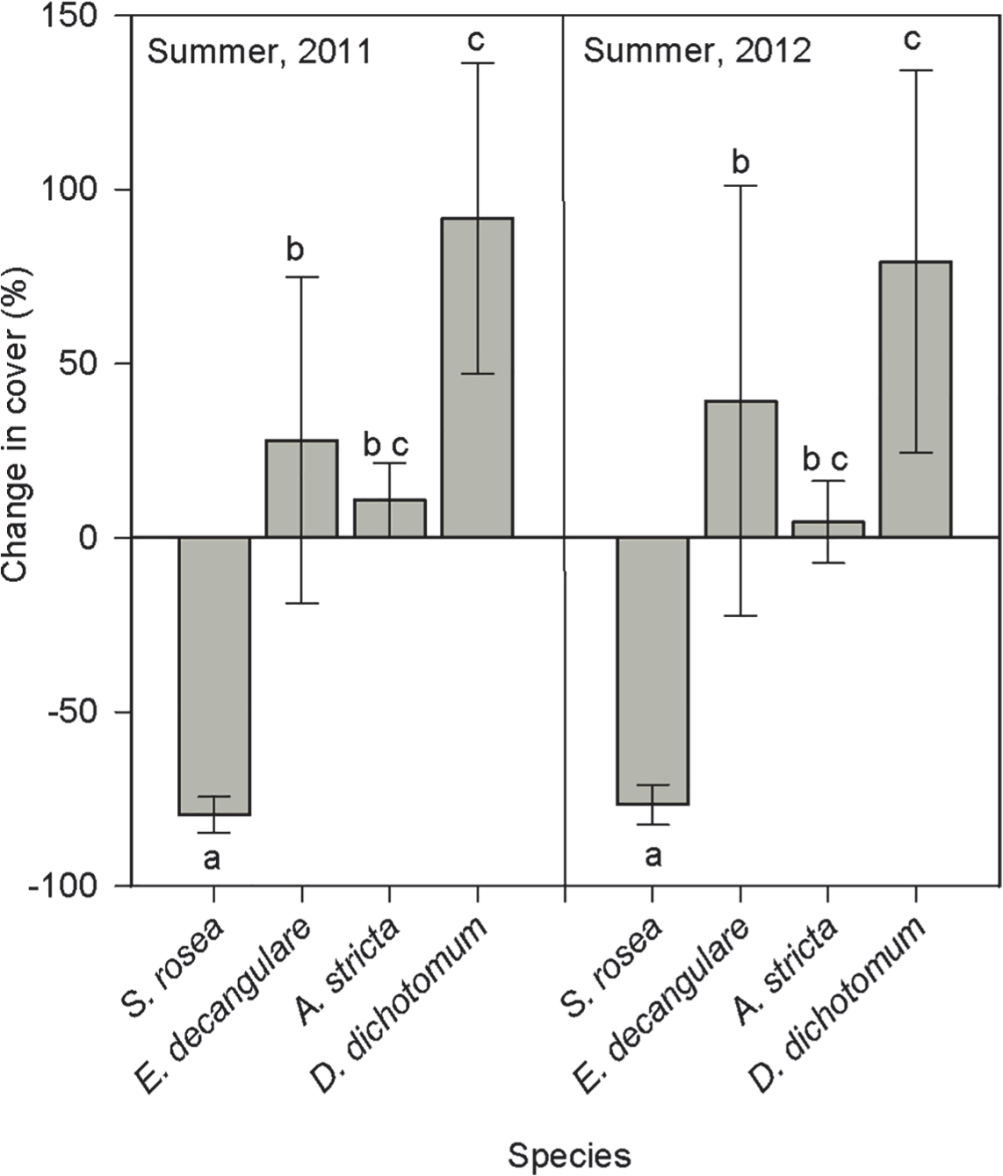
Percent cover change comparisons between *Sarracenia rosea* and three common species. Bar graph comparing percent change approximately one year and two years after treatment applications in 2010. Bars with the same letter are not significantly different (Tukey’s multiple mean comparison test).

In contrast to *S*. *rosea* and the significant treatment indicator species, the majority of the remaining community members appear resilient to the effects of storm surge and able to withstand the addition of fire and drought as well. In fact, when comparing S, J’, H’, and λ across time and treatments, there were no significant treatment effects, but there were significant time effects. Despite there being a fire and a drought the following year, species richness significantly increased over time (F_2, 33_ = 10.84, p < 0.001) and species evenness significantly decreased over time (F_2, 33_ = 7.17, p = 0.003) in all the plots. The species comparisons further revealed that some of the more common species experienced significantly different changes in percent cover than *S*. *rosea* one and two years after the beginning of the study (as indicated by a significant species effect at the end of the first year (F_3, 41_ = 10.70, p < 0.001) and at the end of the second year (F_3, 41_ = 7.20, p < 0.001)). The perennial grasses, *Aristida stricta* and *Dichanthelium dichotomum*, and the perennial herb, *Eriocaulon decangulare*, actually increased in percent cover in both years, while *S*. *rosea* decreased in cover (Fig 2).

## Discussion

### Storm Surge Treatment Effects on *Sarracenia rosea*

Much has been published on the potential impacts of sea level rise on wetlands directly adjacent to the coastline [4, 29–32], but very few studies have considered the impacts of salt water and sediment carried by storm surge floods on plant communities farther inland [15, 33]. This is the first study that experimentally examines the effects of storm surge and sedimentation on plants in GCPP bog assemblages. Our results indicate that even ephemeral spikes in salinity can have pronounced and negative effects on *S*. *rosea*. It was nonetheless surprising to see no indication of elevated soil salinity levels in response to the treatments because Tate and Battaglia [15] found that SWC remained elevated for up to six months in an upland pine savanna after being experimentally flooded with salt water. Since GCPP bogs within pine savannas tend to have higher water tables than the surrounding area [3], we speculate that the extra salt from the flood waters were quickly diluted after treatment application. The area also received approximately 180 mm of precipitation within the twenty-three days before the first post-treatment SWC measurements were taken [20], perhaps further diluting the salt solution. Since acute salt water flooding did not appear to leave lasting effects on the GCPP bog soils in this experiment, the observed declines in *S*. *rosea* percent cover likely resulted from either brief salt water exposure at the root level or from longer term containment of salt water in the pitchers (note that the pitcher liquid within the salt water treated *S*. *rosea* remained salty to the taste three weeks after treatments (M. Abbott, personal observation)). Regardless of the exact cause, our study shows that the effects of salt water flooding on *S*. *rosea* can last through the end of the growing season in which the storm surge occurred.

Although the results suggest that there is an effect of fresh water flooding on *S*. *rosea* cover, the lack of ‘true control’ plots make it impossible to interpret the statistically significant ‘fresh water effect’ as truly an effect of the fresh water flooding treatments or a consequence of some outside force (e.g., drought). Nevertheless, we speculate that if the observed flooding effect was indeed a product of our treatments, it can more likely be attributed to mechanical damage from the force of the surge than to temporary exposure to high water levels. The high water tables in GCPP bogs make these systems prone to frequent temporary flooding (e.g., after a high precipitation event); thus, flooding alone, should have minimal effects on *S*. *rosea* percent cover. Regardless of the underlying cause of the ‘fresh water effect’, we can confidently infer that the added salt in the saline water surge treatments amplified the damage and was responsible for the even greater decrease in *S*. *rosea* cover in the salt water treated plots.

These results superficially appear to conflict with observations made by Goddard et al. [34] after 1 m deep storm surge flood waters covered a GCPP bog in southern Mississippi for several hours after Hurricane Katrina made landfall in 2005. Interestingly, it was mentioned that a population of *S*. *rosea* appeared to be unaffected by the surge [34]. It is important to note, however, that under natural conditions, the extent to which a storm surge affects *S*. *rosea* may be in part determined by hurricane rain patterns. The tremendous amount of rainfall that accompanied Hurricane Katrina (~80 mm reported in nearby Mobile, AL [35]) likely diluted the salt water within the *S*. *rosea* immediately after the surge flooding receded and prevented any negative effects from taking place. Nevertheless, some tropical storms may have relatively little rainfall accompanying them, leaving the salt deposits undiluted until the area is exposed to the next rainfall event [36]. In such cases where little rainfall does accompany a storm surge, salt within the *S*. *rosea* may remain at a highly concentrated state and eventually kill the plants.

### Storm Surge Treatment Effects on Plant Community Composition

Our research suggests that storm surge can initiate shifts in GCPP bog plant composition. The significant three-way PERMANOVA interaction, for instance, suggested that the different water and sediment treatment combinations result in divergent trajectories in composition among the differently treated plots. However, the seemingly different patterns between treatments were not statistically different, possibly owing to other factors: 1) treatment effects on the overall community may have been mild; 2) pre-existing compositional variability may have led to communities diverging in different ways, despite receiving the same treatments; and 3) the fire and drought further affected assemblage trajectories, possibly leading to partial convergence of trajectories. Despite the complex community compositional responses, there were, however, some distinct species-specific responses to the treatments that were likely responsible for the observed shifts in plant composition (Table 2). *Rhexia lutea*, *Ilex glabra*, and *Anthaenantia rufa*, for instance, proliferated in response to sediment deposition, perhaps as a result of being released from competition when sediments were deposited upon neighboring plants. In contrast, *Rhexia alifanus* and *Dichanthelium dichotomum* were most abundant in fresh water treated plots, indicating that they are negatively affected by salt water exposure. More research is needed to determine if these indicator species will either continue to decline or proliferate over time and if these long term responses translate into greater shifts in plant community composition.

If the treatments were indeed too mild to elicit a response by most of the GCPP bog species, we speculate that rapid dilution of the salt water and preexisting species adaptations to minor soil disturbances prevented what could have been greater species losses. Regarding the lack of salt water treatment effects, the high water table in the GCPP bog likely helped to quickly dilute the salt water treatments. A similar dilution effect was observed by Tate and Battaglia [15] who concluded that the relatively lower salt water flooding effects on a fresh water marsh community was due to the consistently higher moisture levels in the marsh soils. With regard to the lack of sediment treatment effects on most of the plant species, GCPP bogs are exposed to small scale soil disturbances frequently enough (e.g., via burrowing animals such as pocket gophers, gopher tortoises, crawfish, etc.) that deposits as deep as those in this experiment may have little effect on plant community composition. Simkin [37] found that vigorous resprouting from under newly deposited pocket gopher mounds in a pine savanna prevented the species composition on the mounds from differing significantly from the surrounding unburied community. Pine savannas—which share many species in common with GCPP bogs—contain a high proportion of perennial species (91%) making them more resilient to sediment burial than other systems that contain greater proportions of annual species without persistent root systems [37]. Unlike wrack deposits, which appear to be impenetrable by many inland species’ resprouts [15], sediment burial by storm surge may be a more ephemeral threat to the majority of plant species in GCPP bogs.

## Conclusion

Overall, the results from this study suggest that a single storm surge can have a significant impact on multiple species—especially *S*. *rosea*—in GCPP bog communities. The majority of the community members, however, appear resilient to the disturbance, suggesting that total species loss from a single storm surge would likely be small. Unfortunately, though, the limited temporal scope of this study and the occurrence of the fire and drought make it difficult to make definitive conclusions about the long term effects. We recommend that future similar studies examining the impacts of storm surge on coastal plant communities strive to do so for a longer period of time (i.e., > 2 years). If it turns out that the biotic legacies of a storm surge disturbance persist for even a few years, then that could be sufficient time for exposure to another hurricane-induced storm surge before populations can fully recover. Thus, we also suggest examination of the effects of repeated storm surges on long term plant community composition and inclusion of fire and drought treatments to determine if these stressors have a synergistic effect. Incorporating fire and drought into the experiment would be an interesting addition because just as the frequency of intense tropical storms is predicted to increase [8–11], it is predicted that with climate change there will be an increased frequency of extreme summer droughts and higher temperatures resulting in elevated evaporation in the northern Gulf Coast region [6, 38–40]. Consistent with the disturbance-mediated competition hypothesis [41], we hypothesize that species more resilient to the combined effects of these disturbances could be given an immediate size and competitive advantage over the sensitive species in GCPP bogs. *Sarracenia rosea*, along with other sensitive species, could thus be driven out of the community by the more resilient species if these disturbances begin occurring together more frequently.
